# The Effectiveness of Emotional Freedom Techniques for Depressive Symptoms: A Meta-Analysis

**DOI:** 10.3390/jcm13216481

**Published:** 2024-10-29

**Authors:** Ji-Woo Seok, Jaeuk U. Kim

**Affiliations:** 1 Digital Health Research Division, Korea Institute of Oriental Medicine, 1672, Yuseong-daero, Yuseong-gu, Daejeon 34054, Republic of Korea; suk6124@kiom.re.kr; 2 KM Convergence Science, University of Science and Technology, Daejeon 34113, Republic of Korea

**Keywords:** emotional freedom techniques, depressive symptoms, meta-analysis, meta-regression, psychotherapy, effectiveness

## Abstract

**Background**: Emotional Freedom Techniques (EFT) have gained attention as a potential therapy for reducing depressive symptoms. However, the evidence remains inconsistent. This meta-analysis aims to assess the overall efficacy of EFT in treating depressive symptoms and explore moderators influencing its effectiveness. **Methods**: A meta-analysis of 18 randomized controlled trials (RCTs) was conducted, with depressive symptom reduction as the primary outcome. Meta-regression explored moderators such as the EFT format, duration, age, and depression severity. **Results**: The analysis showed a significant overall effect size of 1.268 for EFT in reducing depressive symptoms. A moderator analysis revealed that group-based EFT interventions were more effective than individual ones, and participants with moderate depression experienced the greatest benefits. Additionally, shorter interventions were found to be highly effective. **Conclusions**: EFT effectively reduces depressive symptoms, particularly in group settings and for those with moderate depression. Shorter, well-structured interventions may enhance treatment efficiency. Further studies should explore long-term effects and broader applications.

## 1. Background

Depression is one of the most common mental health issues worldwide, leading to severe consequences such as diminished quality of life and an increased risk of suicide [1]. According to the *Diagnostic and Statistical Manual of Mental Disorders, 5th edition* (DSM-5), major depressive disorder (MDD) is diagnosed when an individual experiences at least five of the key depressive symptoms, such as persistent sadness, loss of interest, fatigue, difficulty concentrating, and changes in sleep and appetite, over a two-week period [2]. In contrast, subthreshold depressive symptoms, which may not meet the full criteria for MDD, still contribute to significant emotional distress and functional impairment. Both major depressive disorder and subthreshold depressive symptoms are often referred to as “depression” due to their shared impact on emotional and functional well-being [3,4]. This condition can occur in anyone, regardless of age, circumstances, or background. Life events such as physical or sexual abuse, the death of a loved one, financial difficulties, or sudden and long-term physical illnesses can trigger depressive episodes [5]. However, we still have a limited understanding of how to effectively address these negative emotional states.

Antidepressants are commonly used to treat depressive symptoms, but they come with potential side effects and a risk of relapse. Cognitive behavioral therapy (CBT) is also widely used. While both treatments have demonstrated significant efficacy, they are not universally effective for all patients, and cases of treatment resistance have been reported [6]. Therefore, there has been increasing interest in alternative and complementary therapies, highlighting the urgent need for a rapid-acting, effective, and minimally invasive preventive and therapeutic approach. Such treatments could assist in the prevention, alleviation, and management of depressive symptoms, potentially reducing the social and economic burden associated with depression.

Recent studies have explored alternative and complementary therapies for alleviating depressive symptoms and managing stress, including acceptance and commitment therapy [7,8], herbal remedies [9], mindfulness-based cognitive therapy [10], meditation [11], eye movement desensitization and reprocessing [12], and exercise therapy [13]. However, these treatments often require significant time commitments and may be difficult to follow independently, making them challenging for individuals to implement on their own.

Emotional Freedom Techniques (EFT) is gaining recognition as an alternative therapeutic method for addressing a variety of emotional and psychological issues [14]. EFT is a form of energy psychology that combines aspects of CBT and exposure therapy with acupressure by tapping on specific meridian points on the body [15,16,17]. Developed by Gary Craig in the 1990s, EFT is often referred to as “tapping” due to its method of using the fingertips to tap on acupressure points [17]. The technique involves focusing on a distressing thought or emotion while tapping on 12 specific meridian points located on the head, hands, and torso. While doing this, the individual recites statements of self-acceptance, such as “Even though I have this problem, I fully accept myself” [15,18]. This process is believed to reduce the intensity of negative emotions by down-regulating the amygdala, which is involved in the body’s stress response [14,17,19]. Previous studies demonstrate that EFT can quickly diminish the emotional impact of distressing memories and events, allowing the body to rebalance and accelerate the healing process [20]. Recent meta-analyses have demonstrated that EFT is an effective treatment for conditions such as anxiety [17], depression [21], and PTSD [22]. Additionally, randomized controlled trials (RCTs) have reported EFT’s efficacy in treating conditions such as Hwa-Byung (anger syndrome) [23], tension headaches [24], fibromyalgia [25], frozen shoulder [26], specific phobias [27], and food cravings [28]. These studies highlight EFT’s broad applicability across both psychological and physical conditions.

The stimulation of the meridian points in EFT is reported to promote the release of opioids, serotonin, and GABA, while also regulating cortisol levels, a key stress hormone [20]. These neurochemical changes are associated with pain relief, lowered heart rate, reduced anxiety, inhibition of the fight-or-flight response, and regulation of the autonomic nervous system [20,29]. EFT has been scientifically validated as an effective treatment through objective measures such as reductions in salivary cortisol levels [29] and changes in EEG (i.e., Eletroencephalogram) results [30], indicating a physiological impact on stress regulation. These findings support the efficacy of EFT in reducing stress and promoting relaxation on both a biochemical and neurological level. Given its efficacy in improving various psychological and physical symptoms, EFT is easy to learn, quick to apply, does not require specific tools, and is safe for individuals of all ages [16,17,31]. Based on these previous studies, EFT could be a valuable tool for individuals with depressive symptoms to manage their mental health independently. However, the evidence regarding the effectiveness of EFT is inconsistent across studies, and there is a lack of systematic reviews and comprehensive analyses.

A recent meta-analysis suggested that EFT may be effective in reducing depressive symptoms [21]. However, the study identified several methodological limitations, such as a lack of randomization, small sample sizes, and the absence of standardized treatment protocols. These limitations may have influenced the estimation of the intervention’s effectiveness. The authors emphasized the need for further research, especially with more rigorous methodologies and comparisons to established treatment protocols to strengthen the evidence base for EFT [21]. Although this meta-analysis provided valuable insights into the potential of EFT, it left certain aspects unexplored. For example, the study did not conduct meta-regression to investigate potential moderators, making it difficult to fully explain the variability in effect sizes across different studies. Additionally, the analysis calculated effect sizes using Cohen’s d, based on pre-post comparisons within the intervention group [21]. This approach may overstate the effects of EFT [32]. To address these gaps, our study applies Hedges’ g, which accounts for both the treatment and control groups. We also include meta-regression analyses to explore the effects of potential moderators, such as differences in EFT protocols, participant characteristics, and study designs.

This meta-analysis aims to provide a comprehensive evaluation of EFT’s effectiveness in treating depressive symptoms. It assesses the overall efficacy of EFT and examines how differences between studies, including variations in participant populations and treatment protocols, may influence these results. By exploring these factors, the study seeks to offer more reliable and valid evidence regarding EFT’s clinical utility and its potential as an alternative treatment for depression.

## 2. Methods

### 2.1. Study Design

This study is a meta-analysis conducted on randomized controlled trials comparing the effects of EFT interventions on depression. The study was carried out in accordance with the Preferred Reporting Items for Systematic Reviews and Meta-Analyses (PRISMA) guidelines. The protocol for the meta-analysis was registered with PROSPERO (the International Register of Systematic Reviews; registration number: CRD42023401981).

### 2.2. Selection and Exclusion Criteria

The specific PICO-SD criteria for the meta-analysis in this study are as follows. The population (P) included individuals who were either diagnosed with depression or considered at risk for depression according to established depression assessment tools. The intervention (I) examined was EFT. The comparison (C) groups consisted of individuals who did not receive any other intervention, including those in control groups such as no treatment, waitlist control, placebo, or treat as usual care. The outcome (O) focused on changes in depression symptoms following the intervention. The study design (SD) was limited to randomized controlled trials.

Furthermore, certain studies were excluded from this meta-analysis based on the following criteria: (1) studies that included patients currently on medication; (2) studies that involved other psychological therapies in addition to EFT as an intervention; (3) studies that did not report outcomes related to depression; (4) systematic reviews, literature reviews, meta-analyses, and case reports; and (5) qualitative studies. The retrieved studies were independently reviewed by two evaluators according to the predefined selection and exclusion criteria.

### 2.3. Data Search

The literature search was conducted independently by two researchers from August 2023 to May 2024. The primary objective was to collect studies that compared the progression of depression symptoms before and after the intervention between the treatment group and the control group, focusing on participants who had received an EFT intervention program. The databases used for the search included PubMed, the Cumulative Index to Nursing and Allied Health Literature (CINAHL), the Cochrane Central Register of Controlled Trials (CENTRAL), and the Excerpta Medica Database (EMBASE), covering the period from each database’s inception until January 2023. Additionally, to ensure a comprehensive literature search, Google Scholar was used, and the reference lists of studies included in prior systematic reviews and meta-analyses were reviewed to identify additional RCTs that met the inclusion criteria of this study.

The search strategy was developed based on the core PICO-SD questions. The search terms included the following: (depression OR depressive symptoms OR major depressive disorder) AND (EFT OR Emotional Freedom Techniques OR Tapping Acupoint) AND (randomized OR random OR randomly OR randomization OR RCT OR RCTs) AND (waitlist OR TAU OR treatment as usual OR no intervention OR CAU OR care as usual). There were no restrictions on the country of publication, participant gender, or race. In addition, the reference lists of identified studies, as well as relevant articles suggested by prior meta-analyses and systematic reviews, were manually reviewed.

### 2.4. Selection Process

The selection process for the retrieved studies followed the PRISMA (Preferred Reporting Items for Systematic Reviews and Meta-Analyses) guidelines. Three researchers independently reviewed the collected studies based on predefined inclusion and exclusion criteria. A list of studies retrieved through the database searches was compiled, and duplicate studies were removed. After removing duplicates, the titles and abstracts were reviewed to determine whether the studies met the selection criteria. The full texts of the remaining studies were then reviewed to select the final studies for inclusion. In cases where there was disagreement among the researchers regarding study selection, discussions were held to reach a consensus.

**Figure 1:**
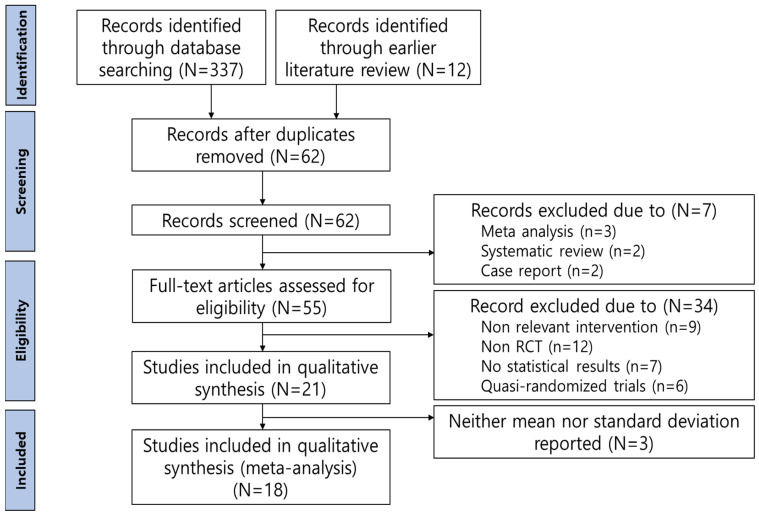
Flow diagram of EFT study selection.

The study selection process is depicted in Figure 1. Initially, a total of 349 studies were identified, with 337 studies sourced from database searches and an additional 12 studies identified through the snowball sampling method. After removing 287 duplicate studies, the titles and abstracts of the remaining studies were screened based on the inclusion and exclusion criteria. Seven studies were excluded because they were either meta-analyses, systematic reviews, or case reports. This screening resulted in the preliminary selection of 55 studies. However, 9 studies were excluded due to the use of adjunctive interventions, such as comparing the effectiveness of EFT with other interventions (e.g., CBT, progressive muscle relaxation, and eye movement desensitization and reprocessing), using combined methods that included both EFT and other interventions (e.g., meditation), or combining EFT with pharmacotherapy. Additionally, 12 studies were excluded for not utilizing a randomized experimental design, 7 studies were excluded for lacking calculable statistical results, 6 studies were excluded for being quasi-experiments, and 3 studies were excluded as, although they provided effect sizes, it was not possible to extract the means and standard deviations for each group before and after the intervention. Finally, 18 studies were included in the meta-analysis to evaluate the effectiveness of EFT interventions across different populations and settings.

### 2.5. Quality Assessment of Included Studies

The critical appraisal of the individual studies selected for the meta-analysis was conducted using the Risk of Bias 2 (RoB 2) tool developed by the Cochrane Collaboration (The Cochrane Collaboration, Copenhagen, Denmark) [33]. Three researchers independently assessed the studies, and any discrepancies were resolved through discussion until a consensus was reached.

RoB 2 is a tool for assessing the risk of bias in RCTs and is composed of five domains: (1) bias arising from the randomization process, (2) bias due to deviations from intended interventions, (3) bias due to missing outcome data, (4) bias in the measurement of the outcome, and (5) bias in the selection of the reported result. The evaluators rated the risk of bias in each domain as low risk, high risk, or unclear based on signaling questions and domain-specific algorithms. The overall risk of bias was classified as low if all five domains were rated as low risk. If there was one domain rated as unclear, the study was categorized as having some concerns. If there were two or more domains rated as unclear or any domain rated as high risk, the study was classified as having a high risk of bias [33].

### 2.6. Data Extraction

In this study, data from a total of 18 selected studies were extracted and coded according to a data extraction form that focused on the characteristics of the participants and the intervention methods (Table 1). The form included information such as the title, author, publication year, participant age, gender, sample size, participant characteristics, type of control condition, duration of intervention, duration per session, total number of sessions, depression measurement tools, and overall depression scores before and after the intervention. When post-intervention scores were reported at multiple follow-up points, only the assessment conducted immediately after the intervention was considered. This approach was employed not only to describe the attributes of each study but also to serve as a basis for analyzing the heterogeneity of effect sizes in subsequent analyses. Additionally, basic statistical data provided by each study, specifically, the means and standard deviations of pre- and post-intervention scores for both groups, as well as sample sizes, were separately compiled.

### 2.7. Statistical Analysis

The analysis was conducted using specialized meta-analysis software including the ”meta” package in the R program and JASP software (ver 0.19.0.0) [50,51]. The statistical analysis for this meta-analysis employed a random-effects model to estimate the overall effect size of EFT on depression outcomes. Hedges’ g was used to assess the effect size, as it corrects for bias, particularly when comparing mean differences between groups [32,52]. The effect size was interpreted using a 95% confidence interval, and the significance was determined if the interval excluded 0 [53].

Meta-regression analyses were performed using the restricted maximum likelihood (REML) method to estimate model parameters [54]. The overall significance of the model coefficients was tested using an omnibus test of regression coefficients [55]. Heterogeneity was assessed using the Q-statistic and I^2^ statistic, where I^2^ represents the proportion of total variability attributable to heterogeneity [56,57]. The publication bias was examined using funnel plot asymmetry, Kendall’s τ rank correlation test, and Egger’s regression test [58,59,60,61].

For more technical details, including the specific statistical formulas and additional assessments such as τ^2^ and τ statistics, refer to the Supplementary Section.

## 3. Results

### 3.1. Characteristics of the Studies

Eighteen studies were included in the meta-analysis, spanning from 2008 to 2023 (Table 1). The participants were primarily adults, including veterans, patients with various medical conditions, and specific demographic groups such as nursing students and adolescents. Sample sizes ranged from 9 to 384, with the most common size category being 26–30 participants per group.

The interventions varied in duration, from single sessions to over 100 sessions, with session lengths ranging from 10 to 90 min. Most interventions lasted several weeks, with EFT delivered either individually or in groups. The control groups included no treatment, waitlist controls, or treatment as usual (TAU). Depression severity ranged from mild to severe, with moderate depression being the most common. Depression was measured using standardized tools, including the Beck Depression Inventory (BDI), the Hospital Anxiety and Depression Scale (HADS), and the Patient Health Questionnaire-9 (PHQ-9). Specific details, including participant characteristics, intervention specifics, and depression measurement tools, are available in Table 1.

### 3.2. Quality Assessment Results

A risk of bias analysis was conducted for the 20 studies using the Cochrane Risk of Bias tool (RoB 2) (Table S1). Of these, 33.33% were rated as having a low overall risk of bias, while the remaining 66.67% were classified as having some concerns or a high risk of bias. The most frequent issues related to insufficient clarity in the randomization process and the management of missing outcome data. Although most studies applied randomization appropriately, a few lacked detailed reporting on allocation concealment, potentially introducing bias. Additionally, 15 studies reported participant dropout rates, ranging from 0% to 40%. In studies with higher dropout rates, many failed to fully account for the potential bias caused by missing data. Outcome measurement and result reporting were generally well managed, with most studies scoring a low-risk level in these areas. However, some studies had unclear blinding of outcome assessment, which could have affected the objectivity of outcome measurements. Full details of the bias assessment for each study can be found in Supplementary Table S1.

### 3.3. Effect Size of EFT

**Figure 2:**
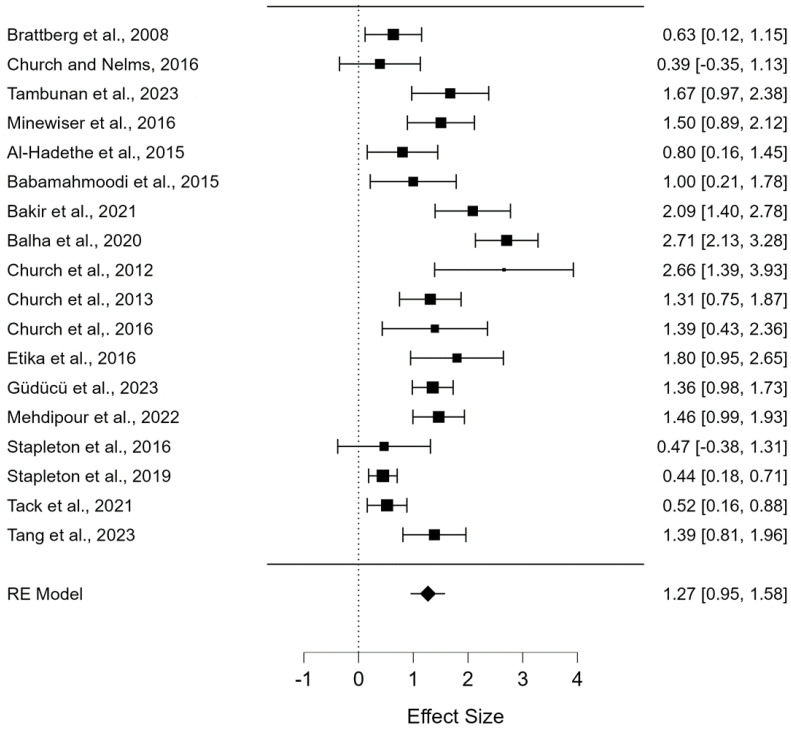
The effect of EFT interventions [25,26,34,35,36,37,38,39,40,41,42,43,44,45,46,47,48,49].

The meta-analysis showed a significant overall effect of EFT on reducing depressive symptoms. The omnibus test of model coefficients indicated that the model was statistically significant (Q(1) = 61.611, *p* < 0.001). There was also significant residual heterogeneity across the included studies (Q(17) = 100.239, *p* < 0.001), with substantial variability in effect sizes. The overall effect size (Hedges’ g) was estimated at 1.268 (z = 7.849, *p* < 0.001, 95% CI: 0.951, 1.585), suggesting a strong impact of the intervention (Figure 2). Further measures of heterogeneity, including τ^2^ and I^2^ statistics, confirmed the presence of high heterogeneity. Detailed statistical outcomes are presented in Table S2.

### 3.4. Moderator Effects of EFT

Meta-regression and subgroup meta-analyses were conducted to explore the sources of heterogeneity in effect sizes across the studies, considering variables such as EFT format (individual and group), control type (no intervention/waitlist and treatment as usual), number of interventions, participants’ age (children/adolescents, adults and over 65 years old), participants’ diagnoses (trauma-related disorders, other psychiatric disorders, chronic physical conditions, and general population or specific groups without chronic diagnoses), and initial depression severity (at risk, mild, moderate, and severe).

The analysis identified significant moderator effects. The omnibus test of model coefficients was highly significant (Q(12) = 64.180, *p* < 0.001), indicating that the combined moderator variables explained a large portion of the variance in effect sizes. Residual heterogeneity was not significant (Q(3) = 3.518, *p* = 0.318), suggesting that the selected moderators effectively captured most of the variability between studies. Fit measures, such as the Akaike Information Criterion (AIC = 29.473) and Bayesian Information Criterion (BIC = 16.854), confirmed a good fit for the model. The I^2^ value was estimated at 16.97%, indicating low heterogeneity among the studies. This suggests that the variability in effect sizes across studies is relatively low, and the overall findings are consistent.

***EFT format.*** The format of EFT interventions had a significant effect on the overall effect size, as revealed by the meta-regression analysis (Estimate = −1.263, *p* = 0.004) (Table 2). This shows that group-based EFT is more effective than individual EFT. In the subgroup analysis, group EFT had a pooled effect size of 1.50, while individual EFT had a pooled effect size of 1.18. These findings suggest that the format in which EFT is administered can lead to notable differences in its effectiveness (Figure S1).

***Total session number.*** The number of sessions had a significant negative effect on the overall effect size (Estimate = −0.029, *p* = 0.008), indicating that a higher number of sessions slightly reduces the effectiveness of EFT (Table 2 and Figure S2).

***Control Type.*** The meta-regression analysis showed that the control type had a significant effect on the overall effect size (Estimate = 1.295, *p* = 0.017), indicating that EFT was more effective when compared to TAU than to no intervention or the waitlist control. In the subgroup analysis, the effect size for TAU was 1.43, suggesting a stronger effect compared to the no intervention/waitlist group (effect size = 1.19) (Table 2 and Figure S3).

***Initial Depression Level.*** The meta-regression analysis indicated that depression severity was a significant moderator, particularly for participants with mild and moderate depression (Table 2). In the subgroup analysis, the effect size for moderate depression was 1.78, indicating a substantial impact of EFT. For participants with mild depression, the effect size was 0.67, while those with severe depression had an effect size of 0.78. Participants at risk for depression had a smaller effect size of 0.62 (Figure S4). These results indicate that the severity of depression plays a crucial role in determining the effectiveness of EFT, with cases of moderate depression potentially resulting in larger effect sizes compared to at-risk groups and mild or severe depression.

***Other moderators.*** Variables such as control type, sample size participants’ age, and participants’ diagnoses, did not show significant effects on the heterogeneity of effect sizes. This implies that these factors may not substantially contribute to the differences observed across the studies included in this meta-analysis (Table 2).

### 3.5. Publication Bias

**Figure 3:**
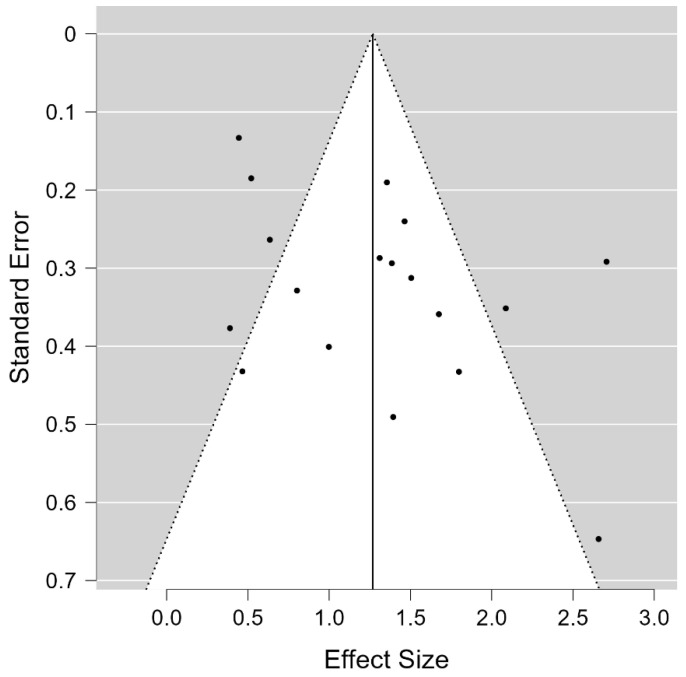
The result of publication bias analyses.

The publication bias of 18 RCTs was estimated. The rank correlation test for funnel plot asymmetry yielded Kendall’s τ = 0.190, *p* = 0.293, and the regression test for funnel plot asymmetry (Egger’s test) was not significant (z = 1.645, *p* = 0.100) (Figure 3). These results suggest that publication bias is unlikely to have significantly influenced the findings. Finally, the trim and fill analysis was conducted to evaluate the impact of potential missing studies on the effect size. The analysis revealed no additional studies were imputed, indicating that publication bias is minimal or absent. Additionally, Rosenthal’s fail-safe N was calculated to be 2002, indicating that a substantial number of additional studies with null results would be required to nullify the observed significance, further supporting the robustness of the results (Figure S5).

## 4. Discussion

This meta-analysis evaluated the effectiveness of EFT in reducing depressive symptoms across different populations and settings. The results showed that EFT significantly alleviates depression, with an overall effect size of 1.268. The analyses for publication bias were non-significant, suggesting that publication bias did not significantly influence the findings. The trim-and-fill analysis did not add any studies, further reinforcing the robustness of the results.

Meta-regression analysis identified key moderators contributing to the variability in effect sizes. The format of EFT played a significant role, with group-based EFT showing greater therapeutic effects than individual sessions. Depression severity also influenced outcomes, with individuals experiencing moderate depression showing the greatest improvements, while those with mild or severe depression had less pronounced effects. Additionally, fewer total sessions were associated with stronger effects. These findings suggest that EFT is broadly effective in managing depressive symptoms, but its impact can be enhanced by tailoring interventions to the EFT format and duration, and the severity of depression.

One key finding was that the effectiveness of EFT varies depending on the severity of depression. Participants with moderate depression showed the most improvement, with a pooled effect size of 1.78, indicating that EFT may be particularly beneficial for this group. While EFT was also effective for those with mild and severe depression, the effects were smaller in comparison. This highlights EFT’s efficacy across all levels of depression severity, with the strongest therapeutic effect observed in those with moderate depression. These results align with findings from other meta-analyses and systematic reviews, which suggest that the effectiveness of non-pharmacological treatments can differ based on depression severity [62,63]. Previous studies emphasize that while such interventions can benefit various levels of depression, patients with severe depression may require more intensive or additional treatments [62,63]. Farah et al. (2016) also highlight that non-pharmacological treatments can be effective alternatives or complements to medication, particularly for patients with moderate depression. This reinforces the need for a personalized approach, where treatment plans are tailored based on the severity of the patient’s symptoms [63].

Another significant finding was that group EFT interventions had a larger effect size (1.50) compared to individual EFT (1.18). This suggests that social interaction and support play a crucial role in enhancing EFT’s effectiveness in a group setting [64,65]. A key benefit of group EFT is the “Borrowing Benefits” phenomenon, where participants gain emotional relief simply by observing others in treatment [19]. Additionally, group settings provide motivation and accountability, encouraging individuals to stay engaged in the therapy [64]. Overall, group EFT appears to offer unique advantages in reducing depression symptoms through enhanced social support and interaction, leading to better outcomes than individual therapy [64,66].

In addition to these findings, our study observed that the effectiveness of EFT tended to decrease as the number of sessions increases (Table 2, Figure S2). This result is consistent with previous research in psychotherapy, including studies on EFT [66,67,68]. EFT is known for its ability to induce rapid emotional change, which may explain why shorter sessions remain highly effective [26]. The success of EFT appears to depend more on the intensity and precision of the intervention rather than the number of sessions. As the number of sessions increases, patients may adapt to EFT or experience a reduction in emotional intensity, resulting in diminishing returns. Previous research has demonstrated that significant symptom relief can occur even after a few sessions [16,42,48,66,69]. For example, Church and Brooks (2010) found that a single 2 h EFT session led to substantial reductions in anxiety, depression, and pain among healthcare workers, with the benefits lasting for 90 days [66]. This suggests that shorter, well-structured EFT interventions can be both time-efficient and cost-effective, providing substantial therapeutic benefits. Therefore, the results imply that extending EFT sessions may not enhance its effectiveness, and shorter protocols may offer optimal outcomes for treating depressive symptoms.

In our study, the results showed that TAU had a greater effect size compared to no intervention or waitlist control groups. This finding is somewhat unexpected because, typically, interventions compared with passive control groups (i.e., no treatment) show larger effect sizes than those compared with active control groups like TAU. One possible explanation is the variability in the quality of TAU [70]. The quality and intensity of care provided under TAU can vary significantly, ranging from minimal services to more comprehensive, high-quality treatment [71,72]. When TAU provides lower-quality or inconsistent care, the difference in effect size compared to no intervention becomes more noticeable [70,73]. This may make it appear as though TAU has a greater impact than it typically would.

Participants’ characteristics, such as age and diagnosis, did not significantly affect the heterogeneity of effect sizes. Consistent with prior research, our findings suggest that these individual differences do not substantially influence the effectiveness of EFT [74]. EFT focuses on emotional regulation by tapping on specific points of the body. This method stimulates both emotional and physical responses. Regardless of individual differences, this approach often triggers similar reactions. As a result, EFT can be widely effective across different groups of people. Therefore, EFT’s universality in addressing emotional issues may explain its broad applicability, regardless of the participant’s specific characteristics.

The findings of this meta-analysis have important practical implications for clinical practice and future research. EFT was shown to be effective across various levels of depression severity, making it a valuable tool for managing depressive symptoms in clinical settings. Given that group-based EFT demonstrated greater therapeutic effects, clinicians can consider utilizing group formats where social interaction and support play a crucial role. Additionally, EFT appears to be particularly effective for individuals with moderate depression, highlighting the need for personalized treatment plans tailored to symptom severity. The results suggest that the effectiveness of EFT does not increase with more sessions, and shorter, more focused interventions may be more effective. This implies that well-structured, shorter EFT protocols could be an ideal approach for treating depression, offering significant practical benefits by minimizing the burden on both patients and healthcare systems. Future research should focus on more rigorous trials involving patients with major depressive disorder (MDD) and on evaluating the long-term effects of EFT through follow-up assessments.

Despite the promising findings, there are several limitations that should be considered. In this study, unexpected results were observed in the comparison between TAU and no treatment. This may be due to the lack of detailed information regarding TAU in the included studies. The specific components of TAU were not clearly described, making it difficult to accurately assess its effects. Future studies should provide more detailed descriptions of TAU to allow for clearer comparisons of its effectiveness. The risk of bias results indicated that some of the included studies had a high risk of bias, particularly in the randomization process and deviations from intended interventions. Missing outcome data and unclear outcome measurement were also identified as factors that could undermine the reliability of certain study findings. These sources of bias may affect the interpretation of the results. To address these issues, future research should employ more rigorous study designs to minimize bias. Improving the transparency and consistency of control conditions like TAU will be essential to enhance the reliability of future findings. Moreover, the sample sizes of the included studies varied widely, with some studies having relatively small sample sizes. In this study, a meta-regression analysis was conducted with sample size as a moderator, and no significant differences in effect sizes based on sample size were found. However, small sample sizes can still limit the generalizability of the findings and may introduce instability in effect size estimates, so the results should be interpreted with caution. Lastly, most studies in this meta-analysis involved participants with subthreshold depressive symptoms, not those diagnosed with MDD, which limits the generalizability of the findings. More RCTs focusing on MDD populations are needed to better understand EFT’s effectiveness for severe depression. Lastly, examining the long-term effects of EFT through follow-up assessments could provide valuable insights into the sustainability of its therapeutic effects.

## 5. Conclusions

This meta-analysis confirms that EFT is effective in reducing depressive symptoms, with a significant overall impact. Group-based EFT and interventions targeting moderate depression showed the greatest benefits. Interestingly, the number of sessions did not enhance EFT’s effectiveness, suggesting that shorter, focused interventions may be more beneficial. While these findings are promising, there is a need for further research, particularly into the long-term effects of EFT and its applications for more severe mental health conditions, such as major depressive disorder. Addressing the limitations related to TAU variability and study bias will also be critical for strengthening the evidence base for EFT as a flexible, accessible, and cost-effective therapeutic intervention.

## Figures and Tables

**Table 1:** The study characteristics of 18 studies selected for the meta-analysis.

Author and Year	Subjects	Age	Groups	Duration of Intervention	Session Number	Depression Level	Depression Assessment
Al-Hadethe et al., 2015 [34]	Male secondary school students with PTSD	16–19	T: Group EFT (n = 20), C: No intervention (n = 20)	2 days/week, 60–90 min/session, 2 weeks	4	Normal	Hospital Anxiety and Depression Scale
Babamahmoodi et al., 2015 [35]	Male veterans with pulmonary injury from mustard gas in Iran–Iraq war	43–58	T: Group EFT (n = 14), C: Waitlist (n = 14)	2 times/day, 90 min/session, 8 weeks	>100	Mild	General Health Questionnaire
Bakir et al., 2021 [36]	Nursing students with premenstrual syndrome	19–22	T: Individual EFT (n = 25), C: No intervention (n = 25)	35 min/session, 9–12 weeks	-	Moderate	Premenstrual Syndrome Scale
Balha et al., 2020 [37]	Patients with substance-related disorders	T: 32.29 (6.4) C: 33.87 (7)	T: Group EFT (n = 45), C: No intervention (n = 45)	1 day/week, 30–45 min/session, 28 weeks	22	Moderate	Symptom Checklist-90-Revised Scale
Brattberg et al., 2008 [25]	Fibromyalgia patients on sick leave	43.8 (8.8)	T: Individual EFT (n = 26), C: Waitlist (n = 36)	7 days/week, 8 weeks	56	Mild	Hospital Anxiety and Depression Scale
Church et al., 2012 [38]	Psychology students	16–18	T: Group EFT (n = 9), C: Waitlist (n = 9)	4 days, 90 min/session	4	Moderate	Beck Depression Inventory
Church et al., 2013 [39]	Veterans with PTSD	T: 49.4 (16.2) C: 54.1(11.1)	T: Individual EFT (n = 29), C: TAU (n = 30)	60 min/session, 4 weeks	6	Moderate	Symptom Assessment-45
Church et al., 2016 [40]	Veterans at risk of PTSD	T: 56 (11) C: 57 (11.9)	T: Individual EFT (n = 12), C: TAU (n = 9)	1 days/week, 60 min/session, 6 weeks	6	Mild	Symptom Assessment-45
Church and Nelms, 2016 [26]	Adults with frozen shoulder	T: 53.88 (7.8) C: 57 (6.27)	T: Individual EFT (n = 16), C: Waitlist (n = 13)	1 day, 30 min/session	1	Normal	Symptom Assessment-45
Etika et al., 2016 [41]	Elderly individuals with mild to moderate depression	60–75	T: Individual EFT (n = 15), C: TAU (n = 15)	1 days/week, 30 min/session, 4 weeks	4	Moderate	Geriatric Depression Scale
Minewiser et al., 2016 [42]	Veterans	T: 50 (15.3) C: 50 (15.1)	T: Individual EFT (n = 32), C: TAU (n = 22)	1 days/week, 60 min/session, 6 weeks	6	Moderate	Symptom Assessment-45
Güdücü et al., 2023 [43]	Women with postpartum depression	T: 30.89 (5.14) C: 29.61 (3.9)	T: Individual EFT (n = 69), C: TAU (n = 67)	15–20 min/session	1–4	Severe	Edinburgh Postpartum Depression Scale
Mehdipour et al., 2022 [44]	Menopausal women	T: 51.77(3.5) C: 52.4(2.85)	T: Individual EFT (n = 44), C: TAU (n = 44)	7 days/week, 90 min/session, 8 weeks	56	Moderate	Beck Depression Inventory
Stapleton et al., 2016 [45]	Adolescents with eating disorders	14–15	T: Group EFT (n = 11), C: Waitlist (n = 11)	1 days/week, 70 min/session, 6 weeks	4	Severe	Depression Anxiety Stress Scales
Stapleton et al., 2020 [46]	People with food cravings and obesity	53 (10)	T: Individual EFT (n = 314), C: Waitlist (n = 70)	4 days/week, 15 min/session, 8 weeks	32	Severe	Patient Health Questionnaire-9
Tack et al., 2021 [47]	Cancer survivors	T: 52 (7.7) C: 52.4 (8.4)	T: Individual EFT (n = 59), C: Waitlist (n = 62)	7 days/week, 10–20 min/session, 8 weeks	56	Mild	Beck Depression Inventory
Tambunan et al., 2023 [48]	COVID-19 patients	-	T: Individual EFT (n = 22), C: No intervention (n = 20)	1 day/week, 30 min/session, 5 weeks	5	Moderate	Patient Health Questionnaire-9
Tang et al., 2023 [49]	Patients with end-stage renal disease	T: 64.25 (5.68) C: 67.65 (7.70)	T: Individual EFT (n = 32), C: No intervention (n = 26)	7 days/week, 15–20 min/session, 12 weeks	>20	Moderate	Hospital Anxiety and Depression Scale

Abbreviation: C, control; PTSD, post-traumatic stress disorder; T, treatment; TAU, treatment as usual.

**Table 2:** Meta-regression analysis of EFT studies on depression.

Predictor Variables	Estimates	SE	Z	*p*	95% Confidence Interval	
					Lower	Upper
EFT format						
Individual	−2.539	1.018	−2.494	0.013	−4.534	−0.544
Total session number (week)	−0.029	0.011	−2.647	0.008	−0.051	−0.008
Sample size	0.005	0.003	1.794	0.073	−54.3	0.011
Control type						
TAU	1.295	0.543	2.384	0.017	0.23	2.36
Depression level						
Mild depression	1.196	0.563	2.124	0.034	0.093	2.3
Moderate depression	0.911	0.408	2.231	0.026	0.111	1.712
Severe depression	−0.067	0.652	−0.102	0.918	−1.345	1.211
Age						
20–65 years old	1.067	0.619	1.724	0.085	−0.146	2.28
Over 65 years old	1.332	0.898	1.483	0.138	−0.428	3.092
Participant diagnoses						
Trauma-related disorders	−1.016	0.641	−1.584	0.113	−2.273	0.241
Other psychiatric disorders	−0.742	0.675	−1.099	0.272	−2.065	0.581
Chronic physical conditions	0.326	0.633	0.515	0.606	−0.914	1.566

## Data Availability

The data supporting the findings of this study are available from the corresponding author upon reasonable request.

## Supplementary Materials

The following supporting information can be downloaded at https://www.mdpi.com/article/10.3390/jcm13216481/s1, Statistical Analysis. Table S1. Risk of bias. Table S2. Summary of statistical outcomes for effect size of EFT. Figure S1. The results of subgroup analysis based on EFT format. Figure S2. Relationship between total session numbers and effect size of EFT interventions. Figure S3. The results of subgroup analysis based on the control group. Figure S4. The results of subgroup analysis based on depression severity. Figure S5. The result of trim and fill analysis Prisma checklist.
