# Supplementary material for: The Effectiveness of Emotional Freedom Techniques for Depressive Symptoms: A Meta-Analysis
Source: J Clin Med. 2024 Oct 29;13(21):6481. doi: 10.3390/jcm13216481 (PMC11547174; doi:10.3390/jcm13216481)
Supplement: Supplementary file 1 [file jcm-13-06481-s001.zip › jcm-3224700-supplementary.pdf]

Statistical Analysis

The statistical analysis for this meta-analysis used a random-effects model to estimate the overall effect size of EFT on depression outcomes.

The analysis was conducted using specialized meta-analysis software including The 'meta' package in the R program and JASP software, ensuring a rigorous and comprehensive evaluation of the included studies [1,2]. Hedges' g was used to assess the effect size, as it corrects for the bias in Cohen's d, particularly when comparing mean differences between two groups [3,4]. Cohen's d often overestimates effect size in small samples, leading to challenges in accurately estimating the population standard deviation. An effect size below 0.15 is small, 0.40-0.74 is medium, and above 0.75 is large [4]. It is interpreted with a 95% confidence interval; if the interval excludes 0, the effect is significant, otherwise, it is not [5]. A narrower interval indicates greater precision, suggesting the estimate is closer to the actual effect [4].

In the meta and meta-regression analyses, the restricted maximum likelihood (REML) method was employed to estimate the model parameters [6]. To assess the overall significance of the model coefficients, an omnibus test of regression coefficients was performed, and the presence of significant effects across studies was confirmed using the Q-statistic and its corresponding p-value [7].

Residual heterogeneity was evaluated using the Q-statistic and I^2 statistics, where the I^2 statistic represents the proportion of total variability attributable to heterogeneity rather than chance. An I^2 of 0% indicates no heterogeneity, less than 25% indicates low heterogeneity, 25% to 75% indicates moderate heterogeneity, and more than 75% indicates high heterogeneity [8]. Additionally, the τ^2 and τ statistics were calculated to estimate the variance and standard deviation between studies [9].

Publication bias was assessed through the asymmetry of the funnel plot, which presents the estimated effect sizes of individual studies on the x-axis [10]. Kendall's τ rank correlation test and Egger's regression test were used to detect the presence of publication bias [11,12]. Furthermore, a Fail-safe N analysis was conducted to estimate the number of missing studies required to nullify the observed effect size, thereby evaluating the robustness of the findings [13].

1. Love, J.; Selker, R.; Marsman, M.; Jamil, T.; Dropmann, D.; Verhagen, J.; Ly, A.; Gronau, Q.F.; Šmíra, M.; Epskamp, S. JASP: Graphical statistical software for common statistical designs. *Journal of Statistical Software* **2019**, *88*, 1-17.
2. Schwarzer, G. meta: An R package for meta-analysis. *R news* **2007**, *7*, 40-45.
3. Schwarzer, G.; Carpenter, J.R.; Rücker, G. *Meta-analysis with R*; Springer: 2015; Volume 4784.
4. Brydges, C.R. Effect size guidelines, sample size calculations, and statistical power in gerontology. *Innovation in aging* **2019**, *3*, igz036.
5. Borenstein, M.; Hedges, L.V.; Higgins, J.P.; Rothstein, H.R. *Introduction to meta-analysis*; John Wiley & Sons: 2021.
6. Partlett, C.; Riley, R.D. Random effects meta-analysis: coverage performance of 95% confidence and prediction intervals following REML estimation. *Statistics in medicine* **2017**, *36*, 301-317.
7. Viechtbauer, W. Conducting meta-analyses in R with the metafor package. *Journal of statistical software* **2010**, *36*, 1-48.
8. Cohen, J. *Statistical power analysis for the behavioral sciences*; routledge: 2013.
9. Sánchez-Meca, J.; Marín-Martínez, F. Confidence intervals for the overall effect size in random-effects meta-analysis. *Psychological methods* **2008**, *13*, 31.
10. Sterne, J.A.; Harbord, R.M. Funnel plots in meta-analysis. *The stata journal* **2004**, *4*, 127-141.

11. Sterne, J.A.; Egger, M. Regression methods to detect publication and other bias in meta-analysis. *Publication bias in meta-analysis: Prevention, assessment and adjustments* **2005**, 99-110.
12. McLeod, A.I. Kendall rank correlation and Mann-Kendall trend test. *R package Kendall* **2005**, *602*, 1-10.
13. Orwin, R.G. A fail-safe N for effect size in meta-analysis. *Journal of educational statistics* **1983**, *8*, 157-159.

Table S1. Risk of bias.

Study	Bias due to the randomization process	Bias due to deviations from intended interventions	Bias due to missing outcome data	Bias in measurement of the outcome	Bias in selection of the reported result	RoB 2 overall bias
Al-Hadethe et al., 2015	Low	Low	Low	Unclear	Low	Some Concerns
Babamahmoodi et al., 2015	Low	Low	Low	Unclear	Low	Some Concerns
Bakir et al., 2021	Low	Low	Low	Unclear	Low	Some Concerns
Balha et al., 2020	Unclear	Low	Low	Unclear	Low	High
Brattberg et al., 2008	Low	Low	High	Unclear	Low	High
Church et al., 2012	Unclear	Low	High	Unclear	Low	High
Church et al., 2013	Low	Low	Low	Low	Low	Low
Church et al., 2016	Low	Low	Low	Unclear	Low	Some Concerns
Church and Nelms, 2016	Low	Low	Low	Low	Low	Low
Etika et al., 2016	Low	Low	Low	Unclear	Low	Some Concerns
Minewiser et al., 2016	Low	Low	Low	Low	Low	Low
Güdücü et al., 2023	Low	Low	Low	Unclear	Low	Some Concerns
Mehdipour et al., 2022	Low	Low	Low	Unclear	Low	Some Concerns
Stapleton et al., 2016	Low	Low	Unclear	Low	Low	Some Concerns
Stapleton et al., 2019	Low	Low	High	Low	Low	High
Tack et al., 2021	Low	Low	Low	Low	Low	Low
Tambunan et al., 2023	Low	Low	Low	Low	Low	Low
Tang et al., 2023	Low	Low	Low	Low	Low	Low

Randomized controlled trials (RCTs) were evaluated with the Cochrane risk-of-bias tool for randomized trials (RoB 2). The proposed judgement about the risk of bias arising from each domain is generated by an algorithm and is expressed as low, high or some concerns.

Table S2. Summary of Statistical Outcomes for Effect Size of EFT

Test	Value	p-value	Confidence Interval (95% CI)	
			Lower	Upper
Omnibus Test (Q-statistic)	61.611	< .001	-	
Residual Heterogeneity (Q-statistic)	100.239	< .001	-	
Effect Size (Hedges' g)	1.268	< .001	0.951	1.585
τ^2	0.358	-	0.158	0.978
τ	0.598	-	0.397	0.989
I^2	82.313%	-	62.215%	92.716%
H^2	5.654	-	3.050	13.729

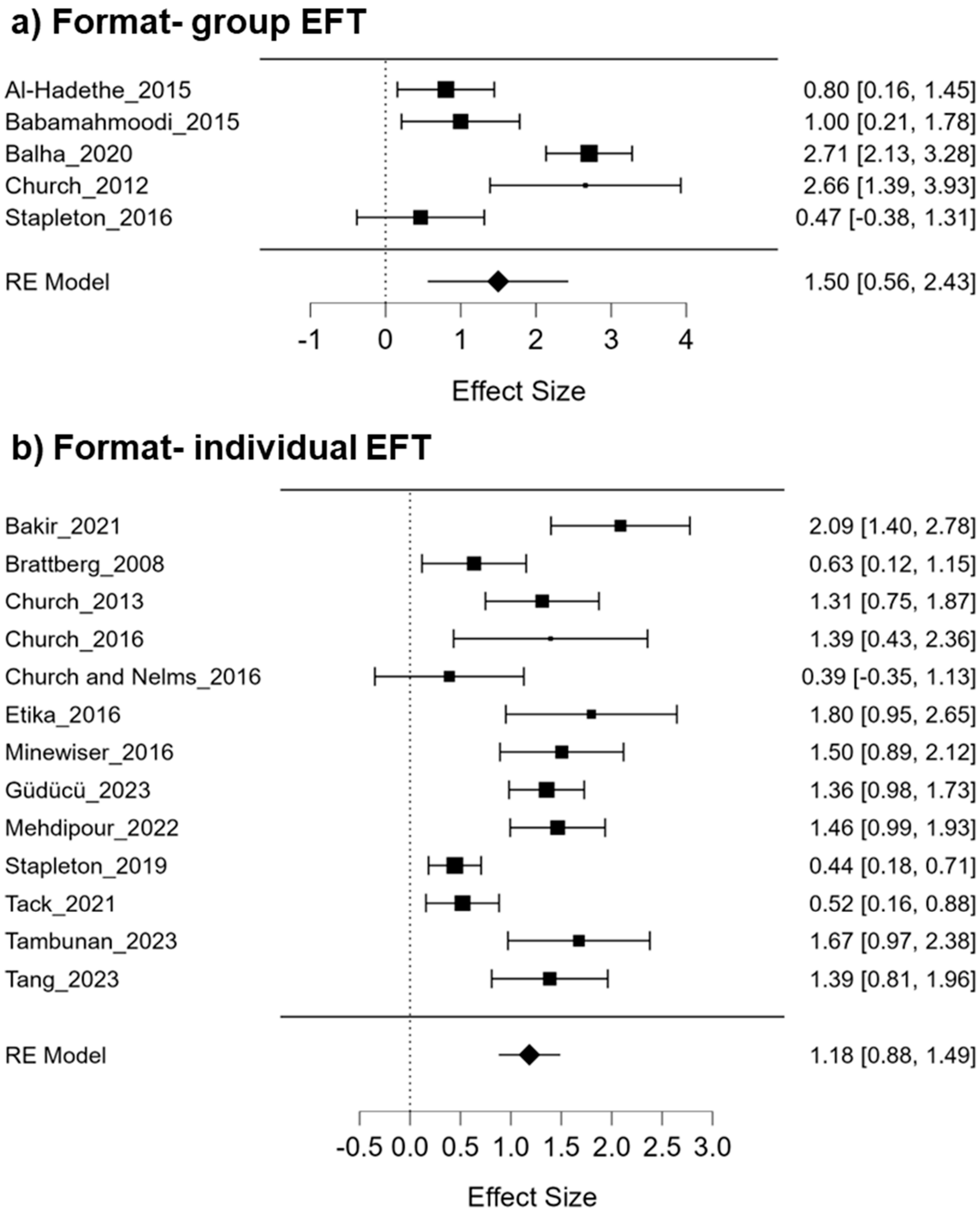

Figure S1. The results of subgroup analysis based on EFT format

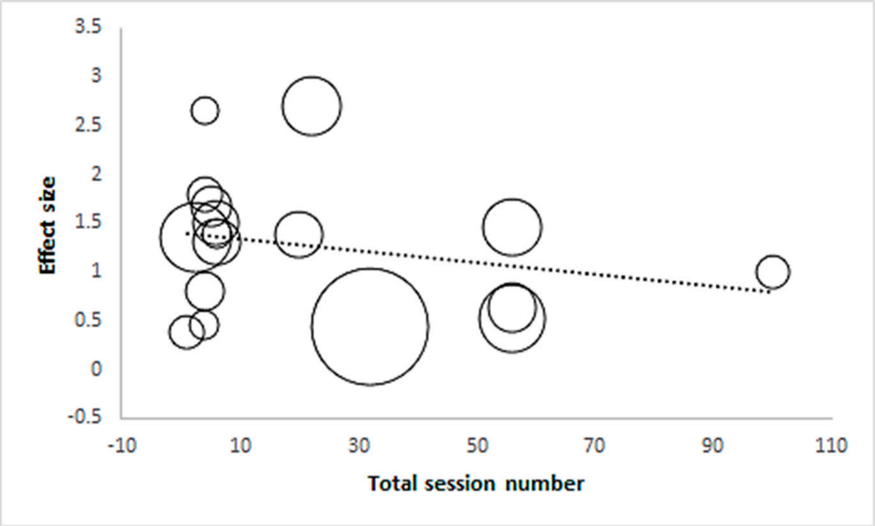

Figure S2. Relationship between total Session number and effect size of EFT interventions

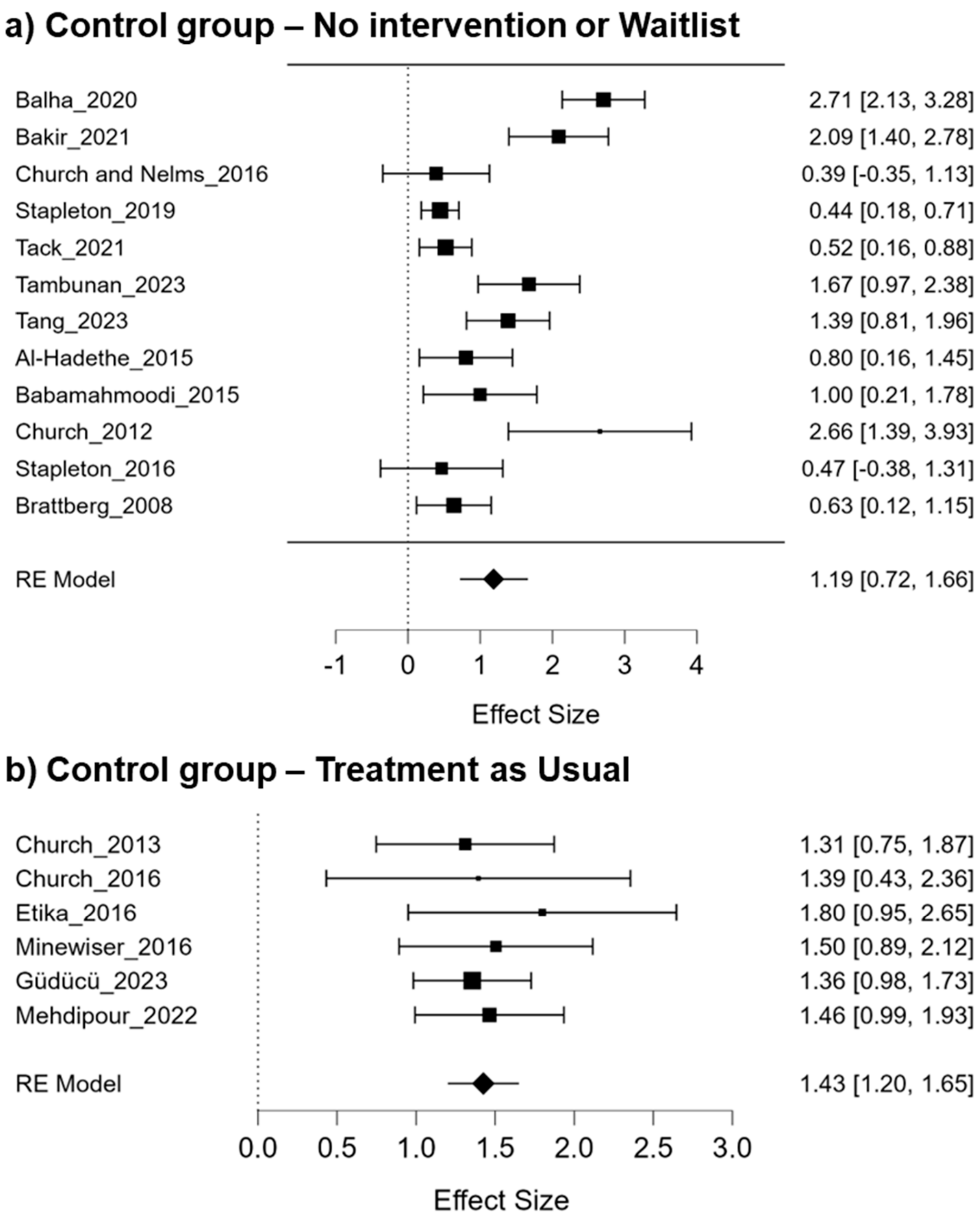

Figure S3. The results of subgroup analysis based on control group

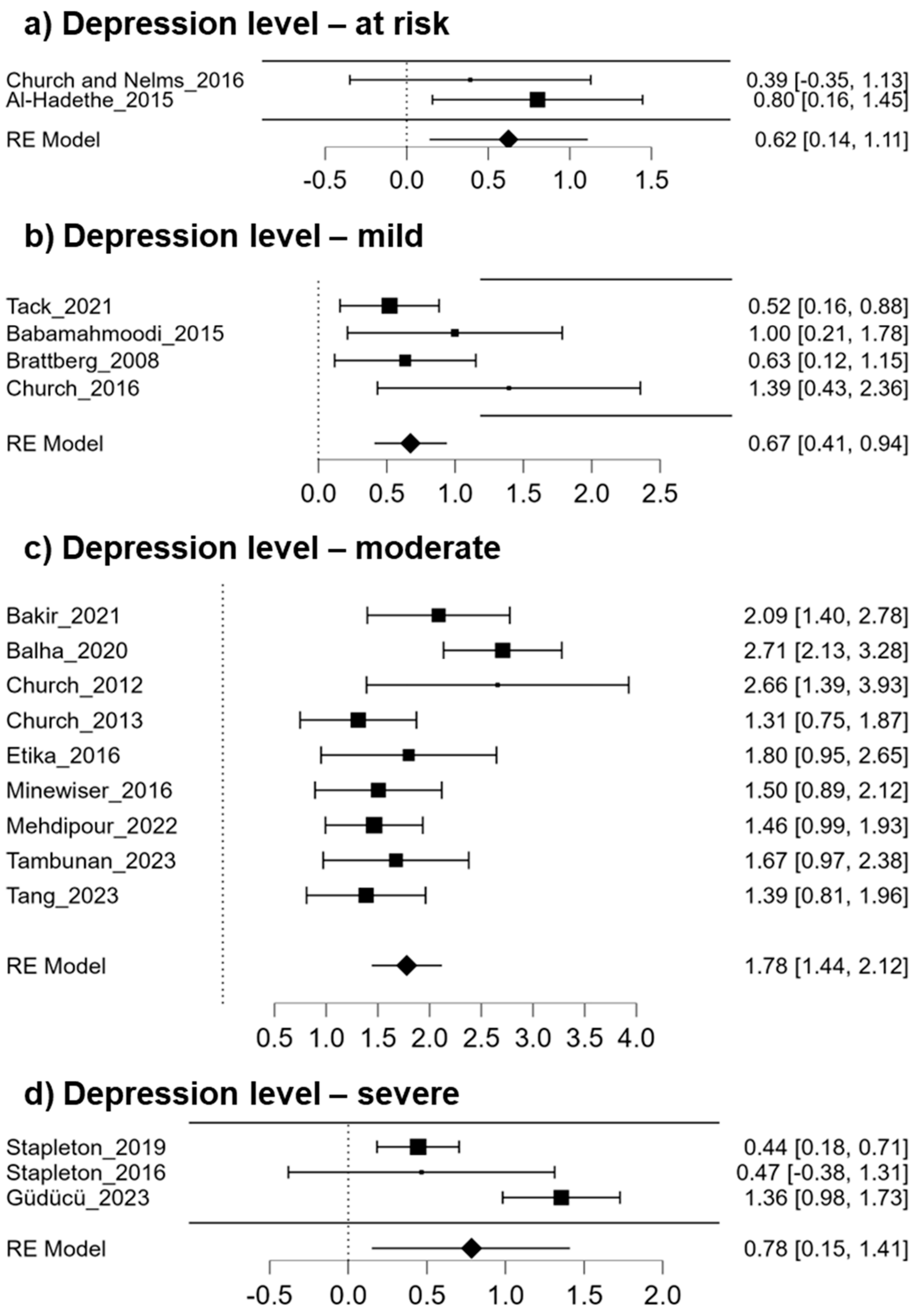

Figure S4. The results of subgroup analysis based on depression severity

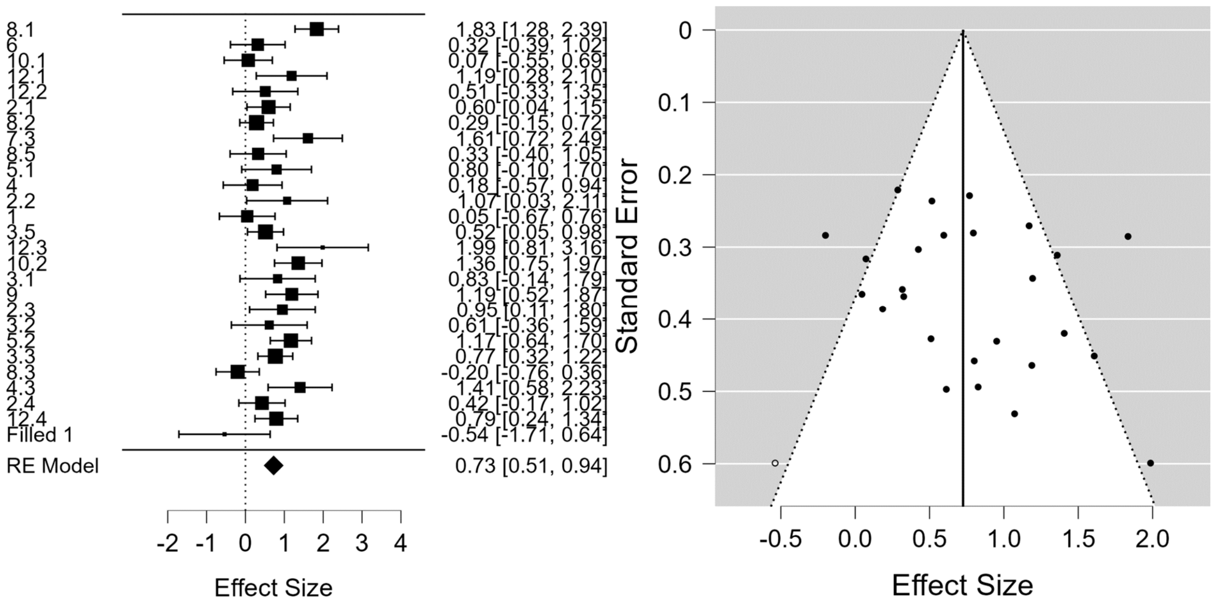

Figure S5. The result of trim and fill analysis
